# Investigation of the possible relationship between the occurrences of atmospheric gravity waves and equatorial Spread-F

**DOI:** 10.1016/j.heliyon.2023.e14151

**Published:** 2023-03-01

**Authors:** A. Olayinka Olawepo, O. Emmanuel Olagunju, O. Victoria Ajani

**Affiliations:** a Department of Physics, University of Ilorin, Nigeria; b Department of Mathematics and Natural Science, William V.S Tubman University, Liberia

**Keywords:** Atmospheric gravity waves, Equatorial spread F, Pre-reversal enhancements, Rayleigh-taylor instability

## Abstract

Equatorial Spread F (ESF), is a manifestation linked to Atmospheric Gravity Waves (AGW). There have not been many studies to ascertain the extensive relationship between the occurrence of AGW and the occurrence of ESF. To evaluate the extent of their relationship, this study used data obtained with the aid of a satellite-borne Atmospheric Infrared Sounder (AIRS) and ionograms obtained using a Digisonde Portable Sounder (DPS-4) located at Jicamarca (geog. Lat. 11.950 ᵒS, long. 76.867 ᵒW and geomagnetic Lat. 2.27 ᵒS, Long. 4.15 ᵒW; Dip 2 ᵒN) during the year 2016. The results also suggest that whenever AGW, under the control of tidal winds, propagates into F-region heights such that both AGW and tidal wind structures act together, it could have great potential at influencing plasma instability growth rate. The result further shows that when AGW has a long enough wavelength to reach the F-region, it tends to influence the factors responsible for the occurrence of the Mixed Spread-F (MSF) type of ESF. MSF is observed to be predominant in occurrence whenever AGW and ESF occur simultaneously compared to the other two types of ESF. The coefficient of correlation between AGW and MSF ranged between 0.1 and 0.5, while for Range Spread-F and Frequency Spread-F, it ranged between ± 0.2. These levels of correlations show that AGW does not directly trigger ESF. The range of the correlation coefficient between AGW and MSF however tends to support the suggestion that AGW occurrence is capable of influencing the factors responsible for a type of ESF occurrence rather than triggering ESF occurrence altogether.

## 1 Introduction

The earth's atmosphere is a cover of gases that, based on temperature, is classified into four main layers. The troposphere is the first layer in the atmosphere having an altitude of 0 km–12 km, ending at the tropopause. The troposphere is closely followed by the stratosphere whose altitude reach is between 12 km and 58 km. The stratosphere ends at the stratopause and is followed by the mesosphere whose altitude is between 58 km and 90 km [1]. The thermosphere is located above 90 km altitude and beyond. Generally, the atmospheric layers of the earth are also stratified as the lower (the troposphere), the middle (stratosphere and mesosphere), and the upper (the thermosphere) atmosphere. The lower atmosphere is the layer of meteorology; the middle atmosphere is the domain of ozone while the upper atmosphere is essentially neutral particles and plasma. Each of these layers of the atmosphere has its different dynamics based on the gaseous composition and types of processes to which these gases are subjected.

Various studies such as [2, 3] have suggested that there exists a connection and interaction among these regions of the earth's atmosphere. As a result, the interaction of the lower atmosphere with the upper layers has become a focus of interest to scientists in a recent study. The upper atmosphere (thermosphere) is composed mainly of the ionosphere. The ionosphere is the layer within the thermosphere where there exist charge ions in significant amounts to influence radio wave propagation. The ionosphere is sub-divided into D (60–90 km), E (90 – 140 km), and F (F1- and F2; 140–500 km) regions each of which supports radio propagation. As a result of the degree of recombination leading to the reduced intensities of the D and E regions during nighttime, the F-region is the most important layer at night, contributing to high-frequency radio propagation. The dynamics of the ionosphere are influenced by electric and magnetic fields and thermospheric wind. The dynamics of the lower atmosphere, are mainly influenced by weather conditions which are determined by several processes, among which is the occurrence of atmospheric gravity waves (AGW). AGW is an important driver of lower atmosphere dynamics and has a substantial influence on weather and climatic conditions [4]. AGW is triggered mostly by meteorological activities sources could be orographic, deep convection, and the adjustment of unbalanced flows near jet streams and frontal systems [5]. AGW is generated in a fluid medium or fluid interface where buoyancy and gravity tend to attain equilibrium, and they propagate mesoscale disturbances, which are capable of transporting energy and momentum in fluid environments. Energy and momentum are been transported from lower to upper altitudes, thereby contributing to atmospheric turbulence. This energy and momentum frequently influence the mean circulation and thermal structure of the middle atmosphere [5]. As AGW propagates vertically and horizontally, it begins to break and form secondary waves which deposit its energy and momentum into the mean flow, resulting in a drag force.

AGW has been shown to infuse into higher altitudes under suitable propagation and atmospheric conditions [6,7,8,9]. Mesosphere and Lower Thermosphere (MLT) thermal structure is significantly influenced by a small drag of AGW through its downward control mechanism [10]. For instance Ref. [11], noted the close correlation between AGW spatial scales in the Mesosphere and Lower Thermosphere (MLT) and plasma bubble scales seen in 6300 Å emissions at the F layer peak. Also, as revealed in the airglow AGW momentum flux analysis by Ref. [7] it showed evidence of AGW spatial and temporal scales and amplitudes in the MLT and extending to the bottomside F layer. Studies by Ref. [12] indicated that AGW could induce plasma dynamics in the ionosphere directly by shifting the plasma in the direction of the geomagnetic field and influencing it towards electric dynamos, either in the F region or in the E region along the magnetic field line. When the electric field traverses the magnetic field a constitutes force is formed that is capable of driving plasma, which results in the formation of vertical plasma drift within the equatorial region of the ionosphere (region ±20ᵒ of the magnetic equator). Plasma drift has consistently influenced the equatorial F layer. The equatorial plasma drift is occasionally upward during daytime and downward at nighttime, and the reversal from upward to downward occurs during the period of 18:00 LT [13].

The main characteristic of the equatorial F region plasma drift is the occurrence of a sharp increase in its upward velocity just before it reverses downward [14]. The Pre-Reversal Enhancement (PRE) of plasma drift is attributed to an enhanced eastward electric field when the E region conductivity decreases instantly after sunset [15,16]. PRE often occurs during the equinox period as soon as geomagnetic field lines are aligned with the sunset terminator, so the eastward polarization electric field becomes strongly close to the sharp horizontal gradient of conductivity [17,18]. PRE behavior and the pattern are determined by solar activity season, longitude, and geomagnetic activity [14]. During post-sunset periods, PRE alters the equatorial F layer to high altitudes, and the bottomside of the F layer becomes denser than the daytime because of the non-existence of photo-ionization at nighttime.

AGWs of different scales and periodicity oscillating into the ionosphere known as traveling ionospheric disturbances (TIDs) are capable of modulating PRE in drift velocity through the wind dynamo [19,20]. The PRE regularly elevates the ionosphere into a higher altitude region, where the collision frequency is lower and more conducive for consistent plasma depletion growth by the Rayleigh-Taylor instability (RTI) mechanism.

**Fig. 1:**
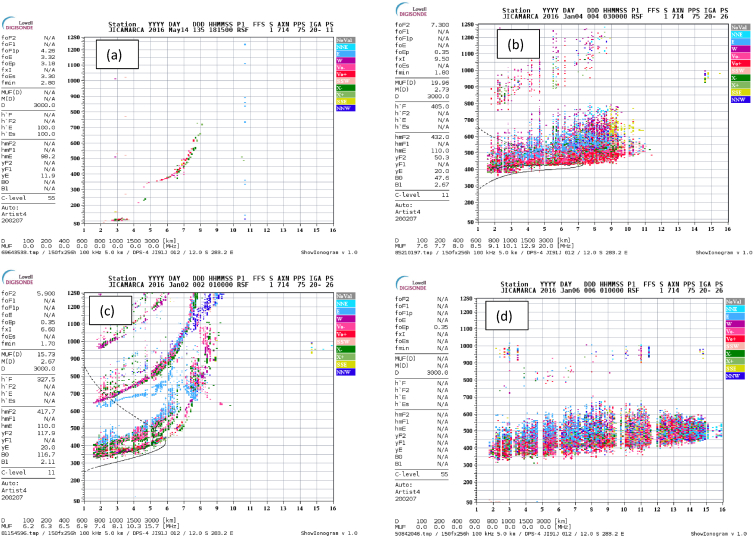
(a–d): Sample ionograms showing three the different types of ESF: (a) No ESF, (b) MSF, (c) FSF and (d) RSF.

Equatorial Spread F (ESF) are observational spread that is seen in ionograms at equatorial regions, which is used to describe plasma instability phenomena that occur in the F-region of the equatorial ionosphere [21]. This phenomenon which was first observed in Ref. [22] was seen as “diffuse echoes” from the F region of the equatorial ionosphere over a wide range of radiofrequency. The ionogram traces, rather than showing a thin line corresponding to the virtual height of the reflecting altitude, as the ionosonde frequency was changed, showed instead a range of virtual heights as if the echoing region were spread over a range of altitudes (range spread, RSF). At times, this spread indicated only at the high-frequency end and looked more like a spread in frequency for a given virtual height (frequency spread, FSF) [23]. The diffuse echoes, suggested to be caused by irregularities in the ionospheric F region, were later named spread F. Consequently, three types of spread F have been identified. Fig. 1(a–d); shows the different types of ESF as it occurs on the ionogram; (a) No ESF, (b) Mixed type of spread F (MSF), (c) Frequency Spread F (FSF) and (d) Range Spread F (RSF) respectively [24]. The Rayleigh-Taylor Instability (RTI) mechanism has been recognized as the basic driver controlling the ESF morphology across different seasons and longitudes [25,26]. According to Ref. [25], three processes could lead to the formation of Equatorial Spread F which are; a linear growth rate of the generalized RTI process, flux tube integrated Pedersen conductivity that controls the nonlinear development, and density perturbations. The R-T Instability is considered to be the main physical mechanism responsible for the growth of equatorial spread F. However, according to Ref. [23], the generalized RTI mechanism is identified to be too slow in explaining the rapid development observed in ESF formation. AGW was therefore proposed as a seeding mechanism for the formation of the irregularity that results in ESF.

This proposition was enhanced by the results obtained from studies carried out by Refs. [19,11] both of which suggested that wave oscillations in PRE were capable of generating strong modulations in the equatorial spread F irregularity process [27]. made an extensive effort to clarify the relationship between PRE and the occurrence of ESF. Where three (3) distinct relationships were identified between them which are namely; the required threshold of PRE for ESF occurrence, linear increase in PRE with an increase in the ESF occurrence probability, and PRE serves as a function of a continuous probability distribution of ESF. Large-scale wave structure (LSWS) development, associated with AGW coupled with the occurrence of shear in zonal drift, was suggested by Ref. [18] and was discovered to be capable of contributing to day-to-day ESF variability. It becomes naturally plausible, therefore, to infer that a solid relationship most likely exists between ESF and AGW as it has been shown in some literature. However, not much study has been done to validate or confirm this relationship. Hence, this paper aims at investigating the relationship between the occurrence of AGW and the generation of nighttime ESF, using data obtained in the American sector of the equatorial ionosphere.

## 2 Data and method

Various studies demonstrated that Advanced Infrared Sounder (AIRS) measurements are particularly suited to validate high-resolution gravity wave occurrence and simulations. The AIRS observations are limited to gravity waves with long vertical wavelengths due to the broad weighting functions of the nadir observation geometry. The rate of the perturbation of the brightness Temperature can be used to determine the temperature amplitude of the gravity waves [28]. Hence, for this study, we have described Brightness Temperature Perturbation (BTP) which was obtained with the aid of AIRS onboard Aqua satellites as proxies for AGW, similar to the method of [29]. This method was used in detail in Ref. [28] where Gravity wave occurrence activity was measured in terms of detrended and noise-corrected 15 μm brightness temperature variances, which are calculated from AIRS channels most sensitive to temperature fluctuations at about 17 km altitudes and above.

ESF occurrence data were obtained by manually observing and identifying ionograms with ESF. The ionograms used were recorded with the aid of a Digisonde Portable Sounder (DPS-4) located at Jicarmaca, (geog. Lat. 11.950 ^ᵒ^S, long. 76.867 ᵒW, and geomagnetic Lat. 2.27 ᵒS, Long. 4.15 ᵒW), an equatorial station in the American-Peruvian sector. BTP data were retrieved from the AIR's data archive at http://datapub.fz-juelich.de/slcs/airs/gravity_waves/data/perturbation. Ionograms were obtained from the Global Ionosphere Radio Observatory (GIRO) portal on DIDbase: https://lgdc.uml.edu/common/DIDBMonthListForYearAndStation?ursiCode=JI91J&year=2016.

Both sets of data were for January to December 2016. This BTP data were used directly as a proxy for the gravity wave data, although one may extract the gravity wave parameter from the BTP data by using a 4th order point polynomial filter [30,31]. for details. This study is interested in the occurrence statistics of AGW rather than its attributes, BTP data suffice as a proxy, hence the use of BTP data. Also, since ESF is known to be a nighttime phenomenon, only BTP falling within the universal nighttime periods of 00:00 UT – 05:00 UT and 18:00 UT – 23:00 UT were considered Nighttime ionograms covering the entire night-time period of the year 2016 were extracted and manually inspected for the occurrence of ESF. Ionograms with ESF were further examined for the ESF type. Thus, ionograms with ESF were grouped into three: RSF, FSF, and MSF.

RSF represents the plasma irregularities in the form of density depletions, which mostly occur at the bottomside of the F-region. Which could be closely associated with large-scale plasma density structures. While FSF usually represents the plasma density irregularities mostly near the F layer peak. MSF is also identified as plasma irregularities with mixed properties of RSF and FSF. RSF, FSF, and with a pattern of different forms of other spread-F types [32, ].

Days and times of occurrences of the two phenomena were noted and recorded. Equations (1)–(4) were used to determine the percentages of occurrence of ESF, types of ESF, and AGW respectively. To determine the occurrence percentage of spread-F (PSF), the total number of spread-F occurrences observed in the ionogram within a day is divided by the total number of ionograms evaluated as expressed in Equ (1). While the percentage type of spread-F (PTSF)was determined by the estimated number of spread-F types such as FSF, RSF, or MSF within the ionogram each day divided by the total number of ionograms for that particular day as shown in Equ (2). The monthly occurrence frequency of AGW was derived by evaluating the total number of AGW within a month and dividing it by the estimated amount of AGW occurrence in 2016 as expressed in equ (3). Equ (4) is used to determine the hourly occurrence of AGW by dividing the estimated sum of the hourly occurrence of AGW within a given time by the sum of the hourly occurrence of AGW. The number of days of the occurrence of AGW and ESF was then statistically analyzed using the method of correlation.

(1) $$Percentage\;of\;Spread\;F\;\left(PSF\right)=\frac{Total\;Number\;of\;ionograms\;with\;Spread\;F\;per\;day\;\left(TNSF\right)}{Total\;Number\;of\;Ionogram\;\left(TNI\right)}\times \frac{100}{1}$$

(2) $$Percentage\;Type\;of\;Spread\;F\;\left(PTSF\right)=\frac{Total\;number\;of\;ionograms\;with\;Spread\;F\;Type\;\left(TSFT\right)}{Total\;Number\;of\;ionogram\;Spread\;F\;\left(TNSF\right)}\times \frac{100}{1}$$

(3) $$\%\;Monthly\;Frequency\;occurrence\;of\;AGW=\frac{Total\;AGW\;occurrence\;per\;month}{total\;GW\;occurrence\;in\;2016}\times \frac{100}{1}$$

(4) $$\%\;of\;the\;hourly\;occurrence\;of\;AGW=\frac{Sum\;of\;hourly\;occurrence\;of\;AGW\;within\;a\;fixed\;time}{Total\;sum\;of\;AGW\;hourly\;occurrence}\times \frac{100}{1}$$

## 3 Results and discussion

### 3.1 AGW occurrence

**Fig. 2:**
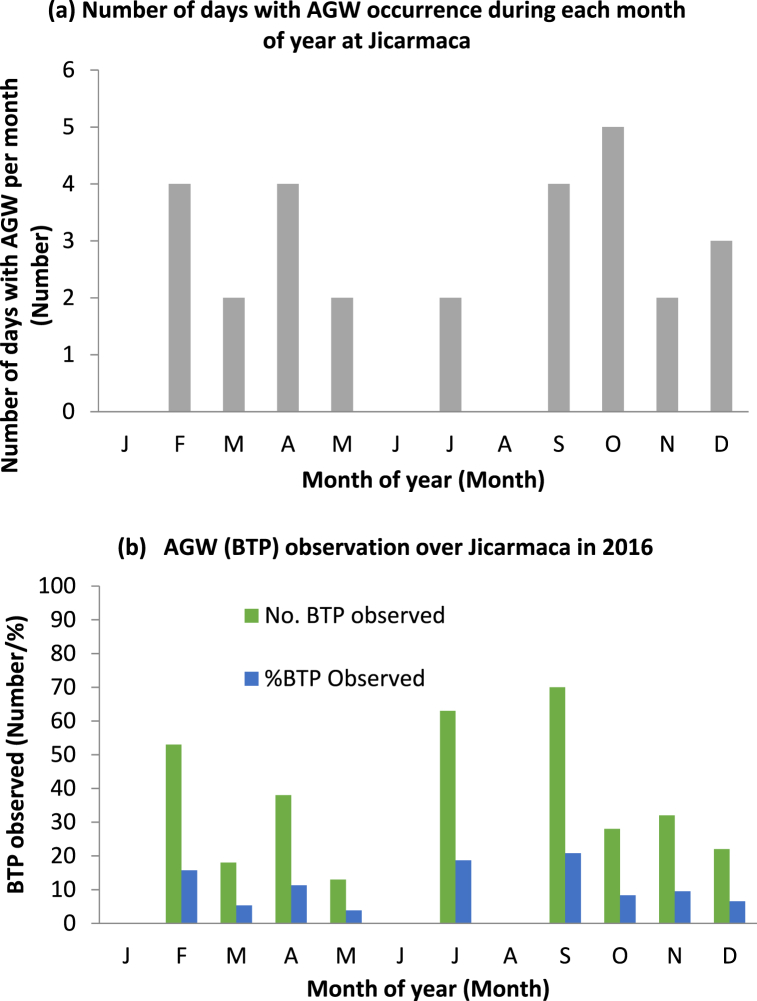
(a–b): Plots showing AGW occurrences at Jicarmaca during 2016. (a) Shows the number of days of AGW occurrence for each month of the year. (b) Number of BTP ((AGW) observed along with the percentage of observation in each month of the year.

The result of AGW observation is shown in Fig. 2(aandb), which reveals the number of days of AGW occurrence for each month and the percentage rate of BTP over Jicamarca. The results show that AGW does not exhibit regular occurrence in the sense that not all the months of the year studied experienced its occurrence. AGW occurred in the months of Feb, Mar, Apr, May, Jul, Sep, Oct, Nov, and Dec. Peak occurrence rate of AGW was in Oct at 18%. Also, it is observed that during the months where there are occurrences, not all the days of the month exhibit the phenomenon. The results show that of the 365 days in the year 2016, only 28 days experienced the occurrence of AGW (Fig. 2(a)). Thus, the percentage occurrence of AGW for the year is 7.7%. Nine of the twelve months of the year had AGW. The months of January, June, and August did not experience any occurrences. Furthermore, for days when there were occurrences, the hourly spread in the occurrence was such that, 72.9% occurrence was observed between 23:00 UT and 05:00 UT (18:00 LT and 00:00 LT) while the remaining 27.1% occurrence was observed between 05:00 UT and 09:00 UT (00:00 LT and 05:00 LT). The months of March, May, July, and November each had two days of AGW occurrences. The month of October with five days of AGW occurrence has the highest number of days. Fig. 2(b) shows the frequency of occurrence of AGW during each of the months along with the monthly occurrence percentages (obtained using equation (3)). The month of September with a total number of 70 observations (within five days of occurrence) has the highest occurrence percentage of 21%. This is followed by July with 63 observations (within two days of occurrence) and an occurrence percentage of 18.7%. The month of December with 22 occurrences (within three days) and an occurrence percentage of 2.5% has the lowest occurrence frequency of AGW in the year under study.

The occurrence percentage of AGW can thus be categorized into three, namely: (i) high, for occurrence percentage greater than 15% (%AGW >15%), (ii) moderate, with monthly occurrence percentage ranging between 8% and 15% (8% > %AGW <15%) and (iii) low level of occurrence with occurrence percentage ranging between 0% and 8% (0% >%AGW <8%). Thus, the months of February, July, and September experienced high AGW occurrences; April, October, and November had moderate occurrences while March, May, and December had low occurrences. There were no occurrences during the months of January, June, and August that did not show the occurrence of AGW. These results further show that the month of July with two days of occurrence but 63 observations has the highest rate of AGW occurrence. The frequency of occurrences for each month is highlighted in Table 1. These results reinforce the fact that AGW does not follow any particular trend in its occurrence.

**Table 1:** AGW observed Monthly occurrences within 2016.

Month	No of Days of occurrence	No of occurrence within days	Frequency of occurrence
January	0	0	0
February	4	53	13.25
March	2	18	9
April	4	38	9.5
May	2	13	6.5
June	0	0	0
July	2	63	31.5
August	0	0	0
September	4	70	17.5
October	5	28	5.6
November	2	32	16
December	3	22	7.3

### 3.2 Equatorial spread F occurrence

**Fig. 3:**
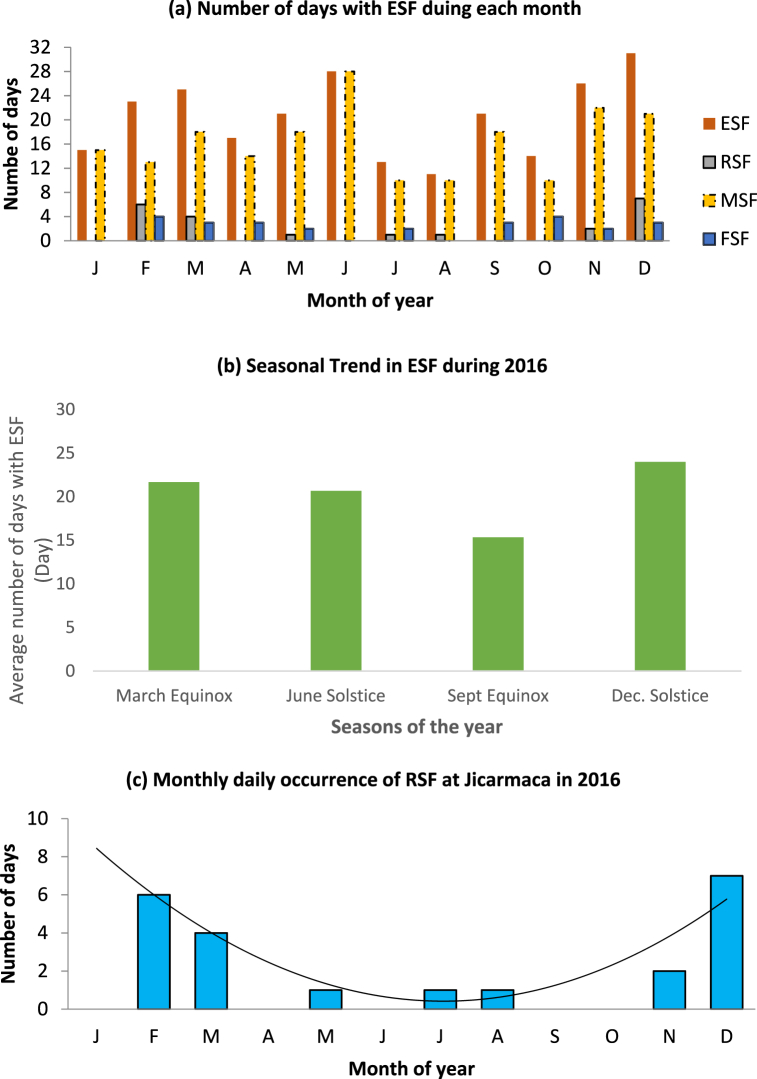
(a–c): Plots showing the occurrence of ESF at Jicarmaca during the year 2016. (a) Relative occurrences of ESF and the three types of ESF (FSF, RSF and MSF). (b) The plot of monthly occurrence of ESF and (c) plot of the RSF type showing the trend during the year.

The occurrence statistics of ESF are shown in Fig. 3(a–c). The plots show that, for the year under study, ESF had a regular occurrence. Fig. 3(a) is the plot of the relative occurrence of ESF and the three types. The plot reveals that, unlike AGW, ESF exhibits a more regular occurrence as it is observed all through the twelve months of the year. The month of December has the highest number of days with occurrence. All the ionograms for the 31 days in the month exhibit ESF. The month of December is closely followed by June with ionograms for 28 out of the thirty days of the month having ESF occurrence. These observations tend to show that ESF occurrence is more prevalent in the solstice months at the station under study. The month of August with 11 days has the least number of days of ESF occurrence. Fig. 3(a) also shows the relative occurrences of ESF types during the year. The plot shows that MSF had an occurrence every month of the year 2016. FSF had an occurrence in eight months of the year, being absent in January, May, June, and August. RSF had an occurrence in seven months (Feb., March, May, July, Aug., Nov., and Dec.) only. Fig. 3(b) shows the seasonal plot of ESF occurrence at Jicarmaca during the year 2016. The y-axis shows the average number of days with ESF observations during each season, while the x-axis displays the seasons. For the seasonal analysis, the months were grouped into four seasons: March equinox (February, March, April); June solstice (May, June, July), September equinox (August, September, October), and December solstice (November, December, January). Although the seasonal plot does not distinctively reveal predominant occurrence during any of the seasons, the trend shows the tendency for the December solstice to have a higher occurrence frequency compared to the other seasons. ESF occurrence is maximum in December solstice with 24 days of occurrence, followed by March equinox with 21.7 days. June solstice had 20.7 days while the September equinox had 15.3 days of ESF occurrence.

Some recent studies that characterize ESF occurrence over Jicamarca reveal its dependent on longitudinal distribution at different seasons which include the difference in the start time, duration, and peak of occurrence. According to Ref. [24], ESF varies with respect to time of day, season, and magnetic dip, An unusual situation could occur where EPB occurrence became high during December (winter) Solstice may be attributed to the closeness of Jicarmaca to the magnetic dip or due to relatively consistent secondary gravity waves, Results from studies such as [33,34] suggest the possibilities of the prevalence of ESF occurrence in the solstice months. Thus, the results observed in this study tend to support the latter submission [35]. observed a significant asymmetric in RSF occurrence distribution during the equinoctial season, while during the solstice seasons, there were cases of discrepancy in the RSF occurrence for the sunset terminator-magnetic field alignment. An irregular pattern of the RSF occurrence percentage across seasons was observed. During the equinox, spread F onset occurs near-vertical drift evening reversal times, while during the December solstice, they occur near the drift reversal times close to solar minimum or as a result of AGW propagation into ionospheric heights [36]. Similar findings were outlined in Refs. [37,38].

Previous studies of EPBs for some stations have established that it exhibits seasonal dependence, being more consistently high during the equinoxes [39,24,40,41]. As highlighted in Ref. [32], FSF ionogram traces represent irregularities near the peak F-region. While RSF ionogram traces represent irregularities located mainly at the bottomside of the F region with an echo that is capable of extending well past the critical frequency of the F region (foF2). Which covers the whole ionosonde frequency range and MSF ionogram traces represent irregularities located throughout the F region.

Fig. 3(a) further shows the plots of the relative occurrence of ESF types. MSF shows a predominance in days of occurrence. MSF type of ESF was observed in all the months with the month of January and June having all the observed ESF being MSF. Thus, MSF seems to be the prevailing type of ESF in this station. FSF type is the next prevalent type of ESF as shown by the plot. However, the number of days of occurrence during each of the months is so small compared to those of MSF. The maximum number of days of occurrence of FSF is 4 (February and October). FSF had an occurrence in all the months except for January and June. RSF occurrence was absent during the months of January, April, June, September, and October. The maximum number of days of occurrence of 7 was observed in December. RSF occurrence is prominent only in three months (February, March, and December). Fig. 3(c) is a plot of the number of days of the occurrence of RSF type of ESF. The plot reveals that RSF monthly occurrence follows a trend over the year (as shown by the polynomial regression line with the regression coefficient of 0.86), having a higher number of days of occurrence during February and March (equinox) and December (solstice) while showing low occurrence during the middle of the year.

The condition for RSF formation occurs when the bottomside depletions reach the topside ionosphere where they are generally classified as starting to peak plumes. In this situation, field-aligned F-region irregularities at the walls of the depletions produce strong coherent backscatter which appears as RSF on the ionograms [42].

The implication of this is that RSF occurrence is more favored in occurrence during some months than others. RSF always has its minimum occurrence around July with two maxima in February and December. The month of December is the month with the highest number of days of occurrence. The time/monthly dependence of the occurrence of RSF is further displayed by the value of the regression coefficient of r which is 0.93 [40]. clearly stated that occurrence rates of each type of Spread F (SF) were strongly dependent on local time, season, and with spread-F occurrence events mostly dependent on the prevailing solar and magnetic activities.

### 3.3 Correlation between AGW and ESF

**Fig. 4:**
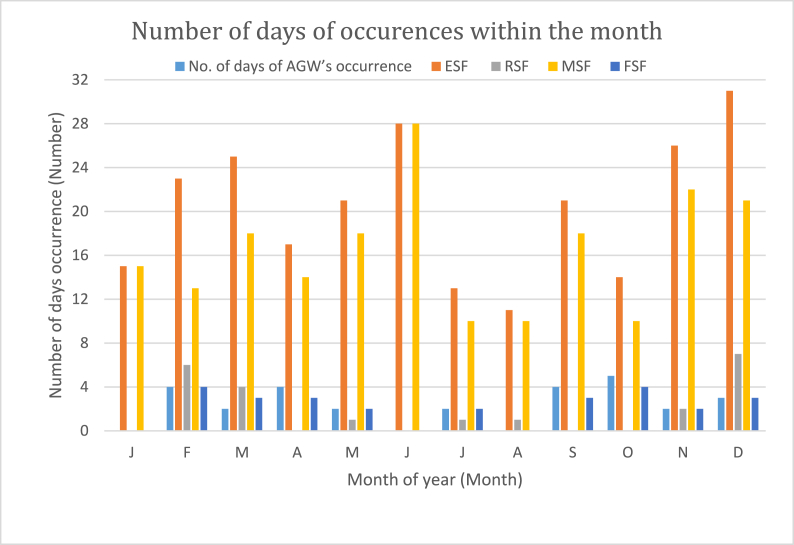
Plot showing the number of days of occurrence of the ESF and the relative occurrences of the three types of ESF at Jicarmaca during 2016.

Fig. 4 is the plot showing the relative number of days of the occurrence of AGW, ESF, and the three types of ESF for each month of the year. ESF, as expected, has regular daily occurrences with all 12 months having between 11 and 31 days of occurrence. The month of August has the least [43] number of days of occurrence while December had ESF occurrence throughout the 31 days (with MSF having occurrence in 21 days). This shows that the seeding mechanism for Rayleigh Taylor's instability is highly favored during these months. All three types of ESF are observed throughout the year except for January and June when only one type of ESF (MSF) was observed. MSF predominates the other two types of ESF throughout the year with the number of days of occurrence ranging between 10 (in July and August) and 28 (in June) per month. In particular, all the ESF observed during January and June (solstice months) are of MSF type. This suggests that the MSF type of ESF is mostly favored (by the prevailing mechanisms) and most likely to be the type of ESF observable under normal circumstances within the Peruvian sector of the equatorial latitude. The number of days of occurrence of FSF ranged between 2 and 4 days per month. FSF type was completely absent in January, June, and August. The RSF type of ESF is observed in seven out of the twelve months of the year. The number of days of occurrence ranged between 1 (August) and 7 (December). Fig. 4 further shows that AGW occurrence is not as frequent as ESF. AGW occurrence was observed in nine months of the year. The months of January, April, and June did not experience AGW occurrence within the period under study. The month of October with a maximum of five days of AGW occurrence has the highest days of occurrence. The months of February, April, and September had four days each with AGW occurrence. All the types of ESF were observed in the months with AGW occurrence except for April, September, and October when there were no occurrences of RSF type. While the least number of days for AGW occurrence is 2 (observed in March, May, June, and November).

**Fig. 5:**
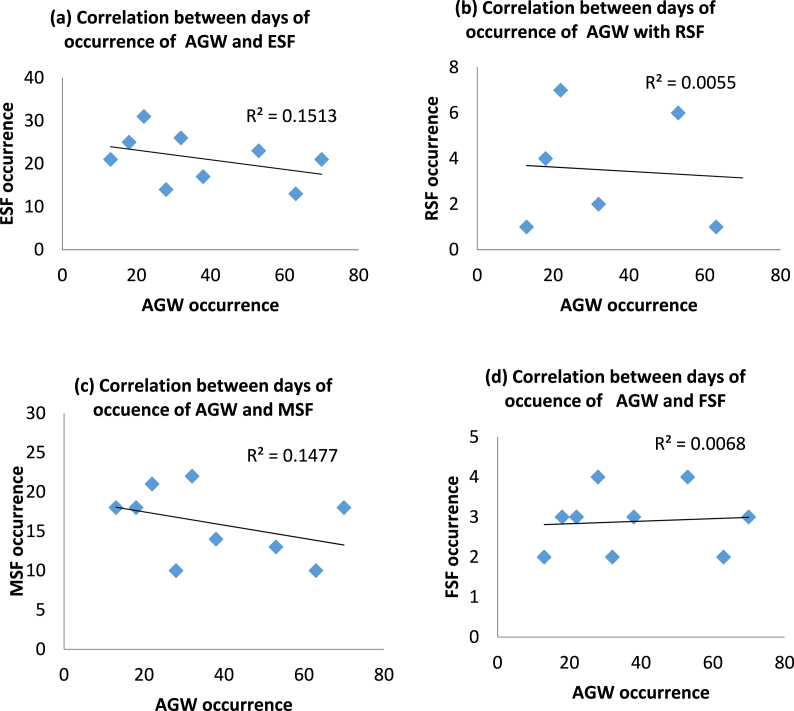
(a–d): Plots showing correlation of the days of (a) occurrence of AGW and ESF and (b–d) occurrence of AGW and the three types of ESF namely RSF, MSF and FSF.

This result rules out the possibility of AGW influencing the occurrence of RSF type. Since MSF has been confirmed to be the prevailing (regular) type of ESF for this station, it means AGW is most likely (if any) to influence FSF. FSF occurred during each month of AGW occurrence. The number of days in which AGW and FSF occurrences coincide is February, May, June, November, and December. These results further suggest a possible relationship between AGW and FSF. Fig. 5 (a – d): shows the correlation plots for the days of the occurrence of ESF (and ESF types) with AGW. The plots show that all except the days of the occurrence of FSF were negatively correlated with the days of the occurrence of AGW. The number of days of ESF occurrence (Fig. 5(a)) shows a negative correlation with days of AGW occurrence with a regression coefficient of 0.1513 (and correlation coefficient, r = 0.39). This suggests that the days of occurrence of ESF and AGW are not significantly related. Among the three types of ESF, only the days of FSF occurrence show a positive relationship with the days of the occurrence of AGW. Fig. 5(b) shows the correlation coefficient between the occurrence of AGW with RSF at 0.0055 (r = 0.07). Fig. 5(c) reveals the correlation coefficient between AGW and MSF to be 0.1477 (r = 0.38). While the regression coefficient of 0.0068 (r = 0.08) is observed (Fig. 5(d)) to exist between the days of occurrences of FSF and AGW. These results show that the days of ESF occurrence do not depend on the occurrence of AGW. To further clarify this, the number of times and the exact time of concurrent occurrences of ESF with AGW were recorded against the type of ESF observable at the time. Concurrent occurrences of AGW and ESF were recorded only on six occasions.

Table 2 shows the months and days (within the period of 18:00–04:00 UT) when the occurrences of the two phenomena coincide and the type of ESF observable at the time. Concurrent occurrences of ESF and AGW were observed during nighttime each in February (5–6), April (20–21), November (28–29), and December (20–21), and two nights in September (26–27 and 28–29). During the period between 18:00 UT and 04:00 UT, a maximum of forty ionograms with ESF occurrences were recorded (ionograms were recorded every 15 min) while a maximum of thirty-one AGW (BTP) occurrences were observed within the same period. The maximum occurrence of AGW was recorded in November.

**Table 2:** Selected Days and Time of concurrent occurrence of AGW and ESF with its observed types within 2016.

Number of observation	Time of A.G.W Occurrences (UTC)	Time of ESF Occurrences (UTC)	ESF type observed
5^TH^ - 6^TH^ February 2016			
1	17.54	18.00	RSF
2	19.45	19.45	RSF
3	19.55	19.55	RSF
4	20.00	20.00	RSF
5	20.06	20.10	RSF
6	20.12	20.15	RSF
7	20.17	20.20	RSF
8	22.12	22.15	RSF
9	22.17	22.20	RSF
10	22.23	22.25	RSF
11	22.29	22.30	RSF
12	22.35	22.35	RSF
13	22.40	22.40	RSF
14	00.42	00.45	RSF
15	00.48	00.50	RSF
16	01.00	01.00	RSF
17	01.05	01.05	RSF
18	03.03	03.05	RSF
19	03.09	03.10	RSF
20	03.15	03.15	RSF
**20**^**TH**^**- 21**^**TH**^**April 2016**			
1	19.37	19.45	MSF
2	19.42	19.45	MSF
3	19.48	20.00	MSF
4	19.53	20.00	MSF
5	19.58	20.00	MSF
6	21.47	22.00	MSF
7	21.52	22.00	MSF
8	21.58	22.00	MSF
9	22.03	22.15	MSF
10	22.08	22.15	MSF
11	23.54	00.00	MSF
12	00.00	00.00	MSF
13	00.05	NAD	NAD
14	00.11	NAD	NAD
15	00.17	NAD	NAD
16	00.22	NAD	NAD
17	02.14	NAD	NAD
18	02.19	NAD	NAD
19	02.24	NAD	NAD
20	02.28	NAD	NAD
21	02.33	NAD	NAD
22	04.19	NAD	NAD
26^TH^ - 27^TH^ September 2016			
1	17.54	18.00	RSF
2	18.00	18.00	RSF
3	20.00	20.00	MSF
4	20.06	20.15	MSF
5	20.11	20.15	MSF
6	20.17	20.30	MSF
7	20.22	20.30	MSF
8	20.27	20.30	MSF
9	20.33	20.45	MSF
10	22.33	22.45	MSF
11	22.38	22.45	MSF
12	22.43	22.45	MSF
13	22.48	23.00	MSF
14	22.54	23.00	MSF
15	23.00	23.00	MSF
16	23.05	23.15	MSF
17	01.07	01.15	RSF
18	01.12	01.15	RSF
19	01.16	01.30	RSF
20	01.21	01.30	RSF
21	01.26	01.30	RSF
22	01.31	01.45	RSF
23	03.33	03.45	RSF
24	03.38	03.45	RSF
25	03.43	03.45	RSF
26	03.49	04.00	RSF
27	03.54	04.00	RSF
28	03.58	04.00	RSF
29	04.03	04.00	RSF
**28th −29th of November**			
1	18.22	18.30	MSF
2	18.27	18.30	MSF
3	18.32	18.45	MSF
4	18.37	18.45	MSF
5	18.42	18.45	MSF
6	18.48	19.00	MSF
7	18.55	19.00	MSF
8	18.58	19.00	MSF
9	19.08	19.15	MSF
10	19.14	19.15	MSF
11	19.19	19.30	MSF
12	21.16	21.30	MSF
13	21.21	21.30	MSF
14	21.26	21.30	MSF
15	21.32	21.45	MSF
16	21.38	21.45	MSF
17	21.44	21.45	MSF
18	21.49	22.00	MSF
19	21.55	22.00	MSF
20	22.00	22.00	MSF
21	22.06	22.15	MSF
22	22.11	22.15	MSF
23	22.17	22.30	MSF
24	00.04	00.15	MSF
25	00.09	00.15	MSF
26	00.14	00.15	MSF
27	00.19	00.30	MSF
28	00.24	00.30	MSF
29	00.28	00.30	MSF
30	00.33	00.45	MSF
31	00.38	00.45	MSF
**28th −29th of September**			
1	19.17	19.30	MSF
2	19.22	19.30	MSF
3	19.27	19.30	MSF
4	19.32	19.45	MSF
5	19.37	19.45	MSF
6	19.45	19.45	MSF
7	19.48	20.00	MSF
8	19.53	20.00	MSF
9	21.58	22.00	MSF
10	22.03	22.15	MSF
11	22.08	22.15	MSF
12	22.13	22.15	MSF
13	22.18	22.30	NSF
14	00.24	00.30	NSF
15	00.29	00.30	NSF
16	00.34	00.45	NSF
17	00.39	00.45	NSF
18	00.44	00.45	NSF
19	00.49	01.00	NSF
20	02.54	03.00	RSF
21	02.59	03.00	RSF
22	03.04	03.15	NSF
23	03.09	03.15	NSF
**20th −21st of December**			
1	20.02	20.15	MSF
2	20.07	20.15	MSF
3	20.12	20.15	MSF
4	21.58	22.00	MSF
5	22.03	22.15	MSF
6	22.09	22.15	MSF
7	22.14	22.15	MSF
8	00.01	00.15	MSF
9	00.06	00.15	MSF
10	00.12	00.15	MSF
11	00.17	00.30	MSF
12	00.22	00.30	MSF
13	02.12	02.15	MSF
14	02.17	02.30	MSF
15	02.22	02.30	MSF
16	02.26	02.30	MSF

*NAD: No available data for ESF.

Concurrent occurrences of AGW and RSF type were recorded twenty times on 5–6 February 2016; AGW occurrence was recorded twelve times with a concurrent observation of MSF type on 20–21 April 2016; MSF was observed fourteen times while RSF was recorded fifteen times on 26–27 September when there were twenty occurrences of AGW; all the thirty-one ESF occurrences observed concurrently with AGW on 28–29 November 2016 were MSF type; on 28–29 September, out of the twenty-three observations of AGW, ESF occurrences were recorded fourteen times with MSF and RSF having occurrences on twelve and two occasions respectively; and on 20–21 December 2016, sixteen occurrences of AGW were recorded with the concurrent occurrence of MSF all the sixteen-time. One major observation from these results is that there were no concurrent occurrences of FSF with AGW throughout. Thus, nullifying the earlier suggestions that AGW could influence FSF. Also, it shows that coincidence in the number of days of occurrences of the ESF and its types cannot be taken for concurrent occurrences. According to Refs. [44,11], where coincidence occurs, the occurrence of AGW is expected to be connected to the occurrence of only two out of the three possible types of ESF, namely RSF, and MSF. it was established by Ref. [44] that Satellite traces (ST) appearing on ionograms are due to oblique reflections from corrugations in the isodensity surfaces which is a result of AGW propagation [45]. In the presence of AGW propagating through the F region, ST is most likely to appear while several minutes later will lead to RSF occurrence.

The results above results tend to favor this assertion as only MSF and RSF were concurrently observed with AGW occurrences.

**Fig. 6:**
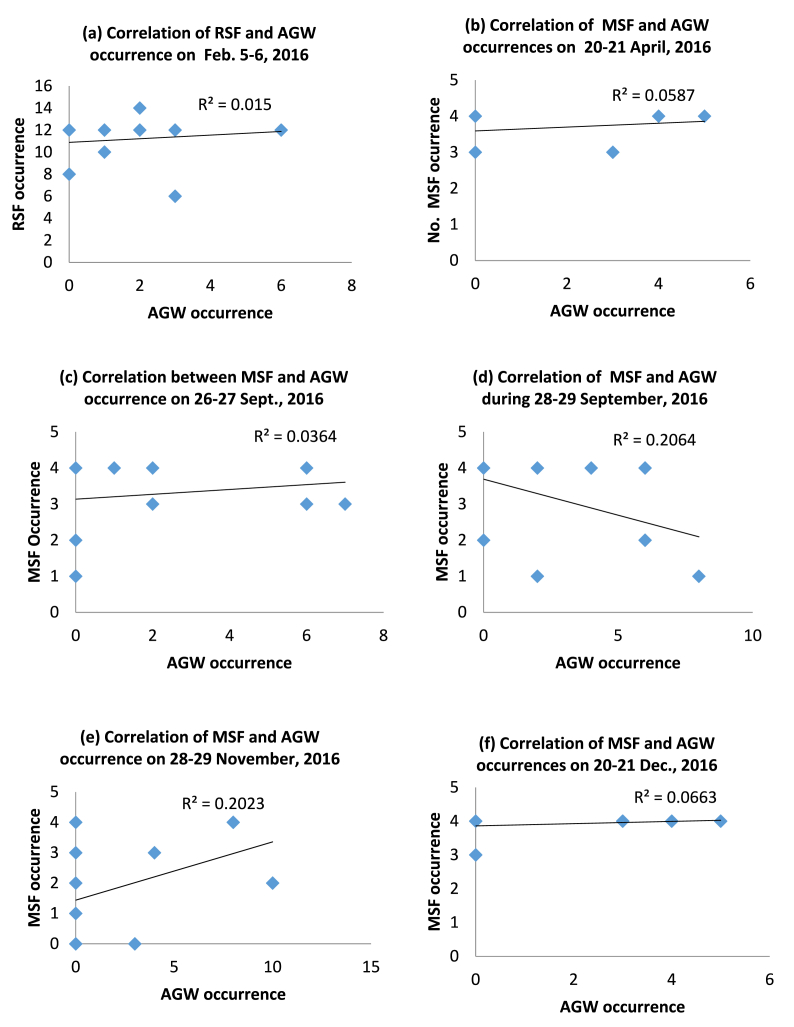
(a-f): Plots showing the correlation of AGW occurrence with the prevailing types of ESF for days where there were concurrent occurrences.

Table 3 is the breakdown of the types of ESF and their percentage of concurrent occurrence with AGW while Fig. 6(a–f) shows scatter plots of the frequency of occurrence of the type of ESF prevailing on each of the days against the frequency of occurrence of AGW for the six occasions of concurrent occurrences. The results show that the occurrence of MSF is more prevalent than RSF on the days of concurrent occurrences of AGW and ESF. MSF occurrence is observed on five out of six occasions. 100% of the ESF observed in April (20–21), September (26–27 and 28–29), and November (28–29), and 75% observed in December (20–21) are MSF. RSF occurrence (100%) is observed only on Feb (5–6). These results imply that the occurrence of AGW tends to favor the occurrence of MSF more than the other two types of ESF since the occurrence of MSF was predominant on days when there were coincidences in occurrences of AGW and ESF.

**Table 3:** ESF types and their percentage of concurrent occurrence with AGW.

S. N	Days (Nighttime) 18:00–04:00UT	No. of times AGW occurred within the period	No. of ionograms with ESF	%RSF	%MSF	%FSF
1.	February 5–6	17	40	100	–	–
2.	April 20–21	15	8	–	100	–
3.	September 26–27	27	40	–	100	–
4.	September 28–29	9	18	–	83	17
5.	November 28–29	31	40	–	100	–
6.	December 20–21	17	40	–	75	25
	TOTAL	116	186

Fig. 6(a–f) shows that, although the correlation between ESF types and AGW frequency of occurrence is positive for all the six methods except for 28–29 September (Fig. 6(d)), the correlation coefficients for all the days were very much less than unity, thus signifying that the degree of dependency of the two phenomena on each other is quite low (if there is any). The regression coefficients as shown in Fig. 6(a, b, c and f) for February (5–6), April (20–21) and September (26–27), and December (20–21) are found to be 0.02 (with a coefficient of correlation, r = 0.14), 0.06 (r] = 0.24), 0.04 (r = 0.2), and 0.07 (r = 0.3) respectively while the correlation coefficient for September (28–29) and November are both 0.2 (r = 0.45) (Fig. 6(e)). These results show there is no significant correlation between these two phenomena.

## 4 Summary and discussion

A statistical study to investigate the relationship between the occurrence of AGW, a lower atmosphere phenomenon, and ESF, a nighttime phenomenon characteristic of the equatorial ionosphere has been conducted. We have used BTP data obtained from AIRS as a proxy for the AGW parameter in conjunction with nighttime ionograms obtained with the aid DPS-4 at Jicarmaca (geog. Lat. 11.950 ᵒS, long. 76.867 ᵒW, and geomagnetic Lat. 2.27 ᵒS, long. 4.15 ᵒW; Dip 2 ᵒN), a station within the dip equator during the year 2016, a year of relatively low solar activity for the study. Results from the study reveal that AGW occurrence is not a regular phenomenon as compared to the occurrence of ESF. ESF is identified as a regular phenomenon emanating from the nighttime irregularity in the equatorial ionosphere. ESF has been established to exhibit diurnal, seasonal, solar cycle, angle of dip, and longitudinal dependence [24,46,40,34,23]. Results from this study show that ESF occurrences at Jicarmaca tend towards being greater during the solstice months than during equinox months. ESF are classified into three types following [24], namely the FSF, RSF, and MSF types. Previous studies have shown that the different types of ESF exhibit time dependence, showing a preference for one season over the other [40]. Results from this present study show that the MSF type of ESF is predominant at Jicarmaca during the year 2016 irrespective of the season. It has been established in section (1) above that the evening PRE is vitally related to ESF and that three different relationships exist between PRE and ESF. These three conditions determine the level of the triggering of the R-T instability mechanism. According to Ref. [47] in addition to PRE, caused by wave structures in plasma density and polarization electric field required to seed the instability, and the F layer bottom side density gradient are important factors in controlling the growth rate of R-T instability. The prominent type of ESF at any period in time may therefore be dependent on any of these conditions. In addition to these, the prevailing type of ESF at any time is been attributed to the sunset time and the time of PRE occurrence [40]. For instance Ref. [24], has shown that MSF is mostly a post-sunset phenomenon while RSF occurs more predominant during post-midnight while [40] reported the onset time of RSF occurrence to be more frequent in equinox than in summer months. Hence the all-time prevalence of MSF as observed in this study may be attributed to a combination of two or more of these factors which are capable of influencing the seeding of the instability.

Unlike ESF, AGW propagation is not locally time-dependent. Results from this study, therefore, confirm works like [48]. According to literature by Refs. [30,49,50], AGW occurrence and extent depend solely on the level of atmospheric convection and other meteorological activities. Also, its direction of propagation could be dictated by planetary waves (PW) which vary from one latitude to the other [48]. Our result shows that AGW (universal night-time) occurrence was recorded at Jicarmaca only on twenty-eight out of the 365 days (universal night-time) of the year under study compared to ESF which is a regular occurrence within the equatorial latitude. This result suggests that the level of atmospheric convention and meteorological factors over Jicarmaca were seldom strong enough during most of the (universal) night-time periods in 2016. Results from this study further reveal that there exists no appreciable correlation in the days of the occurrence of the two phenomena. A correlation coefficient of ∼0.4 was found to exist between days of the occurrence of the two phenomena and even this was not sufficient to conclude their inter-dependency.

Furthermore, not all AGW propagating upward do get to the F-region ionospheric heights. Depending on the wavelengths, AGW is occasionally absorbed in mesopause heights [48]. As a result of the diffusive separation between gas species, it becomes dominant above 100 km. The restoring buoyancy oscillations associated with the atmospheric waves become less viable, so AGW tends to break and get absorbed in this region. However, as observed in this study there are possibilities of AGW propagating successfully beyond the lower ionospheric regions to the F-regions. This, according to Ref. [36] will become possible when AGW achieve large amplitudes enough to reach the bottomside F-layer, with the influence of tidal winds probably controlling the orientation of the AGW thereby causing them to attain the highest altitudes with great effects. The duo effects of the upward movement of AGW and tidal structures acting together have an even more significant potential impact on plasma instability growth rates and plasma bubble seeding. At such periods, the simultaneous occurrence of AGW and ESF may be observed. Also, according to Ref. [47], AGW at the bottomside F-layer altitudes will act to modulate the parameters that are responsible for the growth rates of plasma instability to varying degrees depending on the characteristics of AGW [44,11]. have also observed that, where AGW can reach the bottomside F-layer, it is expected to influence the ionosphere by generating, mainly MSF and RSF types of ESF. The result of this study suggests that whenever AGW reached the bottomside F-layer, it could only contribute to the mechanism responsible for modulating MSF rather than RSF types of ESF. On the six occasions of concurrent occurrences of AGW and ESF, MSF type of ESF was observed to predominate with five of the six days having MSF occurrence. Since the results from this study have shown MSF to be the regular type of ESF (in the absence of AGW), the effect of the penetrating AGW could only be an addition to the background condition for the triggering of MSF. MSF is the type of ESF that combines the attributes of the other two i. e. RSF and FSF. This implies that the influence of AGW at the bottomside F-layer heights is such that it enhances the conditions suitable for the triggering of MSF in preference to the other two types.

## 5 Conclusion

A statistical analysis of the relationship between the occurrences of ESF and AGW has been revealed in this study. We have used AGW data in the form of BTP obtained using AIRS data and ionograms obtained with the aid of a Digisonde Portable Sounder, DPS-4 located at Jicarmaca (geog. Lat. 11.950 ᵒS, long. 76.867 ᵒW and geomagnetic Lat. 2.27ᵒS, long. 4.15ᵒW; Dip 2ᵒN), an equatorial station within the dip equator in the Peruvian sector during 2016. Our results reveal that the occurrences of ESF and AGW are independent of each other. No significant correlation was found between the days of the occurrence of the two phenomena. While ESF is a regular occurrence with a seasonal tendency towards the solstices, AGW propagation neither depends on the local time nor season. The probability of AGW reaching the bottomside F-layer depends on the properties of the wave. In this study, AGW was able to reach F-region ionospheric heights on only six occasions throughout the year under study. AGW with large enough amplitude is unlikely to reach the ionosphere because they tend to be breaking while small-scale gravity waves with favorable conditions could reach the ionosphere and contribute to the seeding mechanism to trigger ESF. The observations of this study suggest that gravity waves without favorable ionospheric conditions themselves might be incapable of triggering ESF. We also found that AGW tends to influence the conditions responsible for the occurrence of the MSF type of ESF which is predominantly a post-sunset phenomenon. The coefficient of correlation between AGW and MSF ranged between 0.1 and ∼ 0.5. These levels of correlations tend to confirm the influence rather than outright triggering of ESF by AGW occurrences.

## Author contribution statement

Olawepo, A. Olayinka, Conceived and designed the experiments; Analyzed and interpreted the data.

Olagunju, O. Emmanuel, Performed the experiments; Wrote the paper.

Ajani, O. Victoria, Contributed reagents, materials, analysis tools or data.

## Funding statement

This research did not receive any specific grant from funding agencies in the public, commercial, or not-for-profit sectors.

## Data availability statement

Data associated with this study has been deposited at Datasets for this research are available at: http://re3data.org:AIRS/Aqua Observations for Gravity Waves; editing status 2020-08-26, re3data.org – Registry of Research Data Repositories. AIRS data are distributed by the NASA Goddard Earth Science Data Information and Service Center (AIRS Science Team Chahine, 2017). Ionospheric data were acquired from Global Ionospheric Radio Observatory (GIRO), http://giro.uml.edu/, the initial DIDBase repository was established with support from the USAF Research Laboratory, (Reinisch and Galkin, 2011).

## Declaration of interest’s statement

The authors declare no conflict of interest.
